# Antitumor Effect of Chalcone Derivatives against Human Prostate (LNCaP and PC-3), Cervix HPV-Positive (HeLa) and Lymphocyte (Jurkat) Cell Lines and Their Effect on Macrophage Functions

**DOI:** 10.3390/molecules28052159

**Published:** 2023-02-25

**Authors:** Bruno Horta, Joana Freitas-Silva, Jani Silva, Francisca Dias, Ana Luísa Teixeira, Rui Medeiros, Honorina Cidade, Madalena Pinto, Fátima Cerqueira

**Affiliations:** 1 Molecular Oncology and Viral Pathology GRP—IC, Portuguese Institute of Oncology of Porto (IPO Porto), Rua António Bernardino de Almeida, 4200-072 Porto, Portugal; 2 ICBAS—School of Medicine and Biomedical Sciences, Port University, Rua de Jorge Viterbo Ferreira, 228, 4050-313 Porto, Portugal; 3 CBQF—Centre for Biotechnology and Fine Chemistry, Associated Laboratory, Higher School of Biotechnology, Portuguese Catholic University, Rua Diogo Botelho, 1327, 4169-005 Porto, Portugal; 4 Interdisciplinary Centre of Marine and Environmental Research (CIIMAR), Terminal de Cruzeiros do Porto de Leixões, Av. General Norton de Matos s/n, 4450-208 Matosinhos, Portugal; 5 AquaValor—Centro de Valorização e Transferência de Tecnologia da Água, Rua Júlio Martins, n°1, 5400-342 Chaves, Portugal; 6 FP-I3ID, FP-BHS, Universidade Fernando Pessoa, Praça 9 de Abril, 349, 4249-004 Porto, Portugal; 7 Faculty of Health Sciences, University Fernando Pessoa, Rua Carlos da Maia, 296, 4200-150 Porto, Portugal; 8 Laboratory of Organic and Pharmaceutical Chemistry, Department of Chemical Sciences, Faculty of Pharmacy, University of Porto, Rua Jorge Viterbo Ferreira, 228, 4050-313 Porto, Portugal

**Keywords:** chalcones, cancer, immunotherapy, tumor associated macrophages

## Abstract

Chalcones are synthetic and naturally occurring compounds that have been widely investigated as anticancer agents. In this work, the effect of chalcones **1**–**18** against the metabolic viability of cervical (HeLa) and prostate (PC-3 and LNCaP) tumor cell lines was tested, to compare the activity against solid and liquid tumor cells. Their effect was also evaluated on the Jurkat cell line. Chalcone **16** showed the highest inhibitory effect on the metabolic viability of the tested tumor cells and was selected for further studies. Recent antitumor therapies include compounds with the ability to influence immune cells on the tumor microenvironment, with immunotherapy being one actual goal in cancer treatment. Therefore, the effect of chalcone **16** on the expression of mTOR, HIF-1α, IL-1β, TNF-α, IL-10, and TGF-β, after THP-1 macrophage stimulation (none, LPS or IL-4), was evaluated. Chalcone **16** significantly increased the expression of mTORC1, IL-1β, TNF-α, and IL-10 of IL-4 stimulated macrophages (that induces an M2 phenotype). HIF-1α and TGF-β were not significantly affected. Chalcone **16** also decreased nitric oxide production by the RAW 264.7 murine macrophage cell line, this effect probably being due to an inhibition of iNOS expression. These results suggest that chalcone **16** may influence macrophage polarization, inducing the pro-tumoral M2 macrophages (IL-4 stimulated) to adopt a profile closer to the antitumor M1 profile.

## 1. Introduction

Cancer continues to be a leading cause of death worldwide, although cancer treatments have improved over recent decades. Most chemotherapeutic drugs act as antiproliferative agents; however, the tumor surrounding microenvironment has an essential effect on the cancer cells’ capabilities [1]. Therefore, searching for new compounds with broad antitumor activity remains an extraordinary challenge. Macrophages are important cells in the tumor microenvironment, designated as tumor-associated macrophages (TAMs) [1,2]. The amount and phenotype of TAMs can influence tumor initiation, progression, angiogenesis, and metastization, promoting disease prognosis [3,4,5,6]. The M1 phenotype, also mentioned as classically activated, is considered pro-inflammatory, and the M2 phenotype, also referred to as alternatively activated, exerts pro-tumoral effects [2]. M1 macrophages produce reactive oxygen (ROS), nitrogen species (RNS), and pro-inflammatory cytokines such as TNF-α, IL-1β, and IL-6 [5,7]. M2 macrophages mainly produce IL-10 and TGF-β [5,7]. Nowadays, reprogramming TAMs into an antitumor phenotype is recognized as one of the antitumor immunomodulation strategies developed in the fight against cancer [2,3,4].

Recent studies revealed that the hypoxic microenvironment of tumors promotes macrophage infiltration [8]. As such, macrophages located at hypoxic regions of the tumor express Hypoxia Inducible Factor (HIF-1), and are able to promote angiogenesis [9] and increasing tumor hypoxia. TAMs’ interference with tumor cells’ metabolism also increases aerobic glycolysis, which is one of the mechanisms responsible for tumor resistance to anticancer immunotherapy [10].

Chalcones are a class of flavonoids recognized for their extensive range of biological activities, including antitumor and anti-inflammatory [11,12,13]. The antitumor effect of chalcones is not limited to apoptosis induction in tumor cells, which makes them promising compounds for cancer therapy [14]. In fact, some chalcones were shown to affect the tumor microenvironment through the modulation of immune mediators released by tumor cells and, therefore, have cancer chemoprevention effects. For instance, some methoxy derivatives of 2′-hydroxychalcone significantly reduced ICAM-1 and IL-8 released by SW480 colon tumor cells [14].

**Figure 1:**
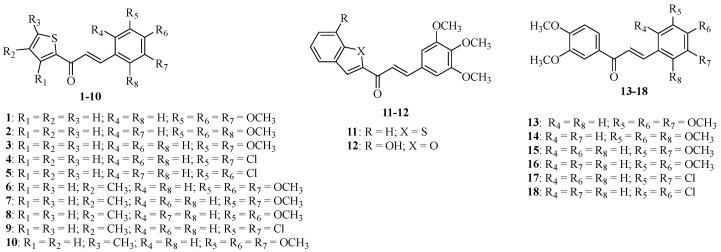
Small library of chalcone derivatives **1**–**18** investigated in this study.

Over recent decades, our research group has identified several chalcones with notable growth inhibitory activity in human tumor cell lines [15,16,17,18,19]. Chalcone derivatives **1**–**18** (Figure 1), in particular, revealed promising antiproliferative activity against A375-C5 (melanoma), MCF-7 (breast adenocarcinoma), and NCI-H460 (non-small cell lung cancer) cell lines, this effect being associated with an antimitotic effect [20]. Aiming to pursue our research on anticancer immunotherapy, namely on their activity on macrophages [21,22,23,24], these chalcones were tested against liquid- and solid tumor-derived cell lines (Jurkat, LNCaP, PC-3, and HeLa). The chalcone that showed the highest antiproliferative effect (**16**) was selected to explore its effect on some human macrophage functions, as the expression of mTORC1, HIF-1α, cytokine characteristics of an M1 (IL-1β and TNF-α) and M2 (IL-10 and TGF-β) profile. The inhibitory effect of the chalcone on NO production by RAW264.7 murine macrophages was also tested.

## 2. Results

### 2.1. Effect of Chalcones on the Metabolic Activity of Tumor Cell Lines

All chalcones were able to affect the metabolic activity of solid- and liquid-derived tumors cell lines, including the cervix HPV-positive tumor cell HeLa, androgen-dependent (LNCaP) and -independent (PC-3) prostate cell lines, and the human lymphocyte cell line Jurkat. All the compounds were able to affect the metabolic viability of the four cell lines tested (Table 1). Doxorubicin was used as a positive control, and the values obtained corresponded to those in the literature [25,26].

For the HeLa cell line, compounds revealed low to moderate inhibition of metabolic activity when tested at 5 μM (9.8–46.8%) and 10 μM (20.3–57.8%), while displaying moderate to high inhibition (26.8–85.8%) at 20 μM. Chalcone **12** with a 3,4,5-trimethoxyphenyl B ring demonstrated the highest capacity for decreasing HeLa cell line metabolic viability (85.8%) at 20 μM. Contrarily, the less active compound was chalcone **18**, possessing chlorine substituents in the B ring (26.8 % inhibition at 20 μM).

Considering LNCaP, the inhibition of the metabolic activity ranged 16.0–37.9% for compounds tested at 5 μM, 28.4–58.9% for compounds tested at 10 μM, and 37.0–78.4% at 20 μM. The highest inhibitory activity (78.4%) was observed for chalcone **7** with a 3,5-dimethoxyphenyl B ring, while polymethoxylated chalcone **17** showed the lowest inhibition (37.0%) at 20 μM.

Against the PC-3 cell line, the metabolic activity inhibition was also depended on the concentration of the compounds tested: 15.4–47.0% for 5 μM; 27.0–64.7% for 10 μM; and 37.0% to 87.6% for 20 μM. Compounds **3**, **10**, and **16**, all with a methoxylated B ring, were the chalcone derivatives that strongly inhibited the metabolic activity of the PC-3 cell (84.6%, 84.7%, and 87.6%, respectively), while chalcone **15** with methoxy groups on both rings showed the weakest inhibition (37.0%).

The metabolic inhibitory effect of chalcones against the non-adherent Jurkat cell line was 6.8–69.4% for compounds tested at 5 μM, 13.0–97.0% when compounds were tested at 10 μM and ranged from 77.3% to total inhibition for compounds tested at 20 μM.

Doxorubicin was used as positive control, and the values obtained were like those reported in the literature [25,26].

Compound **16** with two methoxy groups in both aromatic rings stands out for strongly reducing the metabolic activity of all the cell lines at 20 μM, not demonstrating any selectivity towards any of the adherent cell lines for all tested concentrations (*p* > 0.05). Nevertheless, chalcone **16** at 20 μM is more active in Jurkat than in all the adherent cell lines (*p* < 0.05 for all comparisons).

At 5 μM, chlorinated chalcone **8** resulted in significantly higher inhibition values in LNCaP in comparison to PC-3′s (*p* < 0.05). Chlorinated chalcone **4** was significantly more cytotoxic against LNCaP, while **18** also possessing two chlorine substituents at the B ring demonstrated its highest activity against PC-3 (*p* < 0.05), both at 10 μM. PC-3 revealed a higher sensitivity in comparison to LNCaP for some of the derivatives with methoxylated B rings such as **3**, **10**, and **11** at 20 μM (*p* < 0.05). Chalcones **2** and **4** strongly inhibited the metabolic activity of the HeLa cell line when compared to LNCaP (*p* < 0.05), while **4** and **18** had a weaker effect on the cervix cell line when compared to PC-3 (*p* < 0.05).

In general, when comparing the metabolic inhibitory effect of chalcones possessing the same A ring, those with a 3,5-dimethoxyphenyl B ring showed the most pronounced inhibitory effect, independently of the A ring. Interestingly, most of chalcones with chlorine at B ring displayed a lower activity than those with methoxy groups at the B ring, except for compound **15**.

As compound **16** showed to be the most potent inhibitor of the metabolic activity of all tested tumor cell lines, this chalcone was selected for the study of its effect on macrophage activity modulation and its anticancer potential.

### 2.2. Effect of Chalcone ***16*** on Macrophage Functions

#### 2.2.1. Effect of Chalcone **16** on THP-1 Macrophage HIF-1α, mTORC1 and Cytokine Expression

**Figure 2:**
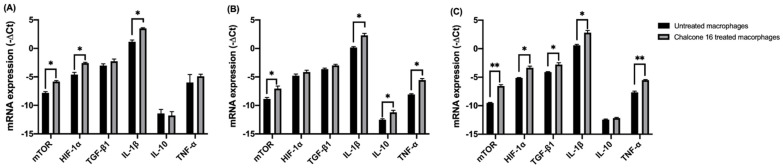
Effect of chalcone **16** on THP-1 macrophage mRNA expression of mTORC1, HIF-1α and cytokines. (**A**) Unstimulated macrophages; (**B**) IL-4-stimulated macrophages; (**C**) LPS-stimulated macrophages. (−∆Cq) of mTOR, HIF-1α, IL-1β, TNF-α, TGF-β1, and IL-10 by THP-1 macrophages. Values represent mean ± SEM (n = 3). * *p* < 0.05, ** *p* < 0.001.

As seen in Figure 2A, for unstimulated macrophages, compound **16** treatment significantly increases the expression of mTORC1 (fold-change = 4.0), HIF-1α (fold-change = 4.1), and the pro-inflammatory cytokine IL-1β (fold-change = 5.1), but no effect is observed on the expression of the other cytokines tested. When macrophages were stimulated with IL-4 (Figure 2B), representing M2 macrophages, chalcone **16** treatment increases the expression of mTORC1 (fold-change = 3.5), but HIF-1α expression, an effector of the hypoxia conditions of tumors, is not influenced. M1 characteristic pro-inflammatory cytokines’ (IL-1β and TNF-α, fold change = 4.5 and 5.7, respectively) expression increased, as well as IL-10 (fold-change = 2.4).

For LPS-stimulated macrophages (Figure 2C) representing the M1 phenotype, an increase was observed for all the parameters tested (mTORC1 fold-change = 7.9; HIF-1α fold-change = 3.4; TGF-β1 fold-change = 2.5; IL-1β fold-change = 4.7 and TNF-α fold-change = 4.4), except for IL-10 expression, which was not affected.

Independent of the stimulation, the treatment of macrophages with chalcone **16** significantly increases the expression of mTORC1.

#### 2.2.2. Effect of Chalcone **16** on NO Production by RAW264.7 Macrophages

The treatment of RAW 264.7 macrophages with 1.3 and 2.5 µM of compound **16** revealed no cytotoxic effect on RAW 264.7 cells (viability of 95.5 ± 3.2% and 98.2 ± 1.3% for 2.5 and 1.3 μM, respectively) (Table 2). The NO inhibition showed a significant decrease (*p* < 0.05) when comparing the activity of chalcone **16** when added at different incubation times after LPS stimulation. Dexamethasone, a known inhibitor of iNOS enzyme production, revealed the same profile. These results suggest that the chalcone **16** mechanism of action is similar to dexamethasone, inhibiting iNOS enzyme production.

## 3. Discussion

In a tumor, along with the cancer cells, immune cells (as macrophages or T-cells) are also present in the tumor microenvironment (TME) [1,27]. The interplay of all the factors of the TME results in the development of the tumor. Macrophages are the majority of immune cells in the TME, and the amount and phenotype of tumor-associated macrophages (TAMs) are determinants for tumor development and progression [2,28]. Briefly, and excluding a variety of subpopulations, macrophages can adopt two opposite phenotypes: M1, or classically activated, with pro-inflammatory activity (producing TNF-α, IL-1β); and M2, or activated, with pro-tumoral characteristics (producing IL-10 and TGF-β) [2,28,29]. TAMs adopt an M2-similar phenotype [2,29]. Docetaxel and tasquinimod are drugs used in cancer chemoimmunotherapy that redirect macrophages to an antitumorigenic M1 profile [1,30,31].

mTORC1 plays an important role in macrophage polarization, mediated by its action at the level of metabolic and inflammatory signaling pathways [32,33]. The effect of mTORC1 on macrophages translates into macrophage polarization to M1, an increase in pro-inflammatory cytokine production by LPS-stimulated macrophages, and suppression of IL-4-induced polarization to M2 [32,33]. In the present study, chalcone **16** treatment induced an increased expression of mTOC1 in either unstimulated, IL-4-stimulated, and LPS-stimulated macrophages.

Usually, the core of the tumor is hypoxic due to anomalous vascularization that implies a deficient oxygen supply [27]. As a response, HIF-1α is activated and acts as a pro-angiogenic factor [28]. HIF influences immune cells, including those associated with tumors [27,28]. Considering TAMS, HIF-1α plays a determining role in the maturation and infiltration of macrophages, as well as in their polarization to an M2 phenotype [27,28]. Chalcone **16** significantly increased the expression of HIF-1α in unstimulated and LPS-stimulated macrophages. However, in IL-4-stimulated macrophages, chalcone **16** did not significantly change HIF-1α expression.

TNF-α and IL1-β are pro-inflammatory cytokines that increase macrophages’ cytotoxicity against tumor cells. They are also characteristic of an M1 macrophage profile [2]. Chalcone **16** significantly increased the expression of IL-1β, independently of macrophage stimulation.

TNF-α expression was only significantly increased for IL-4 and LPS-stimulated macrophages. These results suggest an augmentation in the pro-inflammatory profile of the macrophages, justified by an increase in M1-specific pro-inflammatory cytokines.

With respect to M2-characteristic cytokines, chalcone **16** only exhibited a change in the expression of IL-10 in IL-4-stimulated macrophages. IL-10 is an immunosuppressor cytokine that inhibits the antigen-presenting process and unviable T-cells to recognize and eliminate tumor cells [2,28]. IL-10 levels correlate directly with tumor development [2]. However, chalcone **16** treatment did not cause significant changes in its expression on IL-4 stimulated and unstimulated macrophages when analyzing TGF-β1, another M2-characteristic cytokine that promotes angiogenesis [28]. Only LPS-stimulated macrophages had a significant increase in TGF-β1 expression after chalcone **16** treatment.

NO has dual and controversial effects on cancer, which depend on the type of cancer, concentration, and/or time exposed. In cancers such as prostate, cervical, or melanoma, increased iNOS expression is correlated with a poorer prognosis, while for ovarian and non-small cell lung cancer, increased iNOS expression is considered a positive prognostic marker [34]. M1 macrophages produce high levels of iNOS [1,34]. However, NO production by human macrophages is hard to detect in vitro.

In this study, the RAW 264.7 murine macrophage cell line was used to evaluate the chalcone **16** effect on NO production. The results highlighted the ability of chalcone **16** to inhibit NO production and, by the inhibition profile, a probable inhibitory effect on iNOS expression [23]. These results can reinforce the most likely beneficial effect of compound **16** on macrophage immunomodulation.

Giving special focus to macrophages stimulated with IL-4, which leads to an M2-like macrophage phenotype, treatment with chalcone **16** increased the expression of mTORC1, which induces macrophages polarization to M1, and IL-1β and TNF-α (characteristic of M1). The expression of HIF-1α and TGF-β1, a fulcrum factor for angiogenesis and tumor progression, was not affected by chalcone **16** treatment. However, IL-10 expression increased significantly compared to control cells. Since TAMs are M2-like macrophages, and based on all the results obtained, it was possible to hypothesize that chalcone **16** can influence TAMs, redirecting them to macrophage a phenotype more M1-related. The effect of **16** is also relevant since the M1/M2 ratio in tumors is predictive of disease prognosis [1]. Thus, a higher amount of M2 macrophages is associated with a worse prognosis, while a higher percentage of M1 is associated with a better disease prognosis [1]. Chalcone **16** treatment of IL-4 stimulated macrophages also provoked a significant augmentation in the M1-specific pro-inflammatory cytokines IL1-β and TNF-α. These results suggest a potential chalcone **16** effect on macrophage polarization to an antitumor M1 profile.

## 4. Materials and Methods

### 4.1. Reagents

The acquisition of the reagents and media were as follows: Roswell Park Memorial Institute-1640 (RPMI-1640) medium with Ultraglutamine from Lonza (Verviers, Belgium); fetal bovine serum (FBS) from GE Health Care Life Sciences (GE Health Care, UT, USA), 2-mercaptoethanol from VWR International (Leuven, Belgium); Dulbecco’s Modified Eagle Medium/F-12 Nutrient Mixture (Ham) (DMEM/F-12; 1:1) from Gibco (Paisley, UK); phosphate-buffered saline (PBS) from Fisher Reagent (Geel, Belgium); dimethyl sulfoxide (DMSO) and phosphoric acid from Merk (Darmstadt, Germany); dimethylformamide (DMF) from Romil (Cambridge, UK), TripleXtractor and RNA Kit—Blood & Cultured Cells from GRiSP (Porto, Portugal), recombinant human IL-4 from R&D Systems (Minneapolis, USA). High-Capacity RNA-to-cDNA Kit and Master Mix from Applied Biosystems (Foster City, CA, USA) were used. When not specified, the reagents were from Sigma-Aldrich (ST. Louis, MO, USA).

### 4.2. Chalcone Derivatives ***1***–***18***

Chalcone derivatives **1**–**18** were synthesized and characterized by the Laboratory of Organic and Pharmaceutical Chemistry, Department of Chemical Sciences, Faculty of Pharmacy/CIIMAR research group as previously described [20]. The powered compound was dissolved in dimethyl sulfoxide (Acros Organics) and stored at −20 °C and diluted, before each assay, at the desired concentration, in the appropriate culture media.

### 4.3. Cell Lines and Cell Culture

PC-3 cell lines were obtained from the European Collection of Cell Cultures (ECCAC) and LNCaP from the American Type Cell Culture (ATCC). The other cell lines were kindly provided by Maria José Oliveira (THP-1 and HeLa), Institute for Investigation and Innovation in Health (i3S), Portugal; Henrique Almeida (Jurkat), i3S, Portugal; Maria São José Nascimento (RAW 264.7), Faculty of Pharmacy, University of Porto, Portugal. The complete culture medium for tumor cell lines was composed of RPMI-1640 supplemented with 10% of FBS and 1 μg/mL of gentamicin. In the THP-1 cell line, we supplemented all the culture media described with 2-mercaptoethanol (0.05 mM). RAW 264.7 cell line was cultured in DMEM/F-12 supplemented with FBS and gentamicin. All cell lines were incubated in a humidified atmosphere containing 5% CO_2_, at 37 °C.

### 4.4. Cytotoxic Assay (MTT) for Adherent Tumor Cell Lines

The MTT colorimetric assay was conducted based on the original procedure proposed by Mosmann [35], with modifications [24]. Cell lines (HeLa, PC-3, or LNCaP) were seeded at a concentration of 1.5 × 10^4^ cells/well (96-flat-bottom well culture plate) and incubated for cell adherence (24 h) [36]. After removing the supernatants, the cells were treated with chalcone derivatives, at the desired concentrations, for 48 h [36]. Additionally, included non-treated and doxorubicin-treated cells in the assays as controls [26].

Once the incubation period was over, we washed the cells, and MTT (0.2 mg/mL) was placed in contact with the cells for 4 h at 37 °C in a CO_2_ incubator [21]. The MTT formazan product was solubilized with DMSO while shaking for 10 min, and absorbance was measured 545/630 nm (STAT FAX 3200) [24]. The formula used to calculate the cytotoxicity was as follows:

cellular metabolic viability inhibition (% of control) = 100 − (abs sample/abs control × 100).

### 4.5. Cytotoxic Assay (MTT) for Non-Adherent Tumor Cell Lines

Jurkat cells (50 μL; 1.5 × 10^4^ cells/well) were placed in 96-well plates, and after a 24 h incubation [36], the desired test concentrations of the compounds (50 μL) were added. Several controls were included in the experiments consisting of untreated cells, doxorubicin-treated cells, compound blank, and medium blank. A new 48 h period of incubation was carried out [36], after which an MTT solution 0.2 mg/mL per well was added [21] and left for 4 h in a CO_2_ incubator at 37 °C. After MTT reduction by viable cells, 50 μL of an SDS solution (20% SDS in DMF/H_2_O (1:1)) was added to the wells to dissolve formazan [21]. After reading absorbance, as already stated, cytotoxicity was calculated as follows:

Inhibition of metabolic viability (% of control) = 100 − [(abs sample-abs blank)/(abs control − abs negative control) × 100]

### 4.6. THP-1 Macrophage-Phenotype Differentiation

The human leukemic monocyte cell line THP-1 (1 × 10^6^ cell/mL) was differentiated into macrophages by phorbol 12-myristate 13-acetate (PMA, 0.1 μg/mL) treatment for 72 h [37]. After washing and an additional incubation in the culture medium for 24 h [37,38], an M0 phenotype was obtained. The effect of chalcone 16 on the various macrophage phenotypes was achieved by their treatment for 24 h [39], in three different stimulation conditions: unstimulated, LPS-stimulated (1 μg/mL) [39], and IL-4 stimulated (20 ng/mL) [37]. LPS or IL-4 stimulation was performed simultaneously to chalcone 16 treatment. The control used were non-treated macrophages.

### 4.7. Assay for Quantification of mRNA Expression

For mRNA cytokines expression quantification, THP-1 derived macrophages (M0) were obtained as stated in Section 4.6. The influence of chalcone 16 with cytokine mRNA expression was studied exposing the macrophages to different concentrations of compound 16 for 6 h [37] in three different conditions described above: un-stimulated, LPS-stimulated and IL-4 stimulated. Non-treated macrophages, exposed or not to LPS or IL-4 stimulation, were also used. After incubation, the media were removed and cells were washed. TripleXtractor reagent (GRISP) was used for mRNA isolation, stored at −80 °C until use. The RNA fraction was separated, samples were purified (GRS Total RNA Kit—Blood & Cultured Cells commercial kit) and RNA concentration and purity were assessed using a NanoDrop Lite spectrophotometer (Thermo Scientific^®^, Waltham, MA, USA). For cDNA synthesis, mRNA samples were used (High-Capacity RNA-to-cDNA Kit; Thermo Fisher Scientific). Reactions were carried out in the StepOneTM Plus PCR Real-Time PCR instrument, with 1x Master Mix, 1x probes (TaqMan^®^ Gene Expression assays mTOR, Hs00234508_m1; HIF1α, Hs00153153_m1; TGF-β1, Hs00998133_m1; IL-1β, Hs01555410_m1; IL-10, Hs00961622_m1, and TNF-α, Hs02621508_s1; Applied Biosystems, Foster City, CA, USA). The housekeeping gene used was B2M (TaqMan^®^ Hs99999907_m1; Applied Biosystems, Foster City, CA, USA), which served as an endogenous control to normalize the results. The analysis of the results was conducted utilizing the StepOneTM Software v2.2 (Applied Biosystems, Foster City, CA, USA) with the same baseline and threshold set for each plate, to generate quantification cycle values (Cts) for all the mRNA targets in each sample.

### 4.8. Nitric Oxide Production Assay

RAW 264.7 cells were placed in a 96-well culture plate (1 × 10^6^ cell/mL; 200 µL) and incubated for 2 h to allow the cells’ adhesion [22]. The culture media was then discarded, and an LPS (1.5 µg/mL) and chalcone solution were added at equal volume and added together (0 h). Chalcone treatment was also started 6 or 14 h after stimulation with LPS to evaluate the effect of compound **16** on iNOS expression/activity. Cells were incubated for a total of 24 h after stimulation, and after that period, 100 µL of the culture media were placed into a new 96 flat-bottom well plate, and 100 µL of Griess Reagent (1:1 solution of 1% *w/v* sulphanilamide solution in phosphoric acid (5% *v*/*v*) and naphtylethylenediamide (0.1%) in deionized water) were added to every well. The reaction occurred for 10 min protected from light and at room temperature [22]. The optical density was measured (545/630 nm; STAT FAX 3200), and the effect on nitrite production was calculated as follows:

Inhibition of NO production (% of control) = 100 − [(abs sample-abs blank)/(abs control − abs negative control) × 100]

### 4.9. Statistical Analysis

IBM SPSS Statistics 26.0 for Windows was used. The results were displayed as mean ± SEM. To obtain meaningful results, media ± 2SD was used to perform the statistical evaluation of the effect of chalcone derivatives against the metabolic viability of tumor cell lines. The normality of the distribution of the results was confirmed by the Shapiro–Wilk test of normality, and the homogeneity of variance assumption was checked using Levene’s test. One-way Anova was conducted for the experimental cytotoxicity analysis with Bonferroni’s correction post hoc test. The differences in cytokine mRNA expression were analyzed using the Student’s *t*-test. Statistical significance was considered for *p* < 0.05.

## Figures and Tables

**Table 1:** Effect of chalcones **1**–**18** on the metabolic viability of tumor cell lines expressed as percentage of cellular inhibition (%) compared to control.

Compound	Concentration (µM)	Inhibition of Metabolic Viability (% of Control)			
		HeLa	LNCaP	PC-3	Jurkat
**1**	5	39.1 ± 0.1	16.0 ± 5.4	23.5 ± 7.2	58.6 ± 13.6
	10	33.5 ± 5.6	33.5 ± 6.9	33.6 ± 10.7	90.8 ± 4.2
	20	43.6 ± 5.7	39.1 ± 1.1	47.6 ± 10.5	T.I.
**2**	5	24.0 ± 12.5	24.3 ± 1.9	31.2 ± 4.3	6.8 ± 3.9
	10	22.4 ± 7.5	39.2 ± 5.3	41.9 ± 4.0	20.8 ± 1.6
	20	29.5 ± 4.2	45.3 ± 4.1	45.4 ± 8.7	81.8 ± 7.3
**3**	5	29.4 ± 10.4	23.1 ± 8.5	33.4 ± 5.8	55.3 ± 15.8
	10	20.3 ± 5.7	40.9 ± 5.9	32.0 ± 5.0	87.8 ± 5.6
	20	61.1 ± 10.1	74.4 ± 0.8	84.6 ± 2.7	T.I.
**4**	5	21.9 ± 3.8	30.1 ± 4.9	33.3 ± 4.7	29.1 ± 11.5
	10	29.3 ± 9.7	40.5 ± 2.7	27.0 ± 2.8	66.1 ± 10.9
	20	26.8 ± 4.9	56.3 ± 2.4	52.3 ± 3.9	91.6 ± 5.9
**5**	5	31.3 ± 4.7	31.2 ± 2.1	28.4 ± 9.2	60.2 ± 9.5
	10	33.7 ± 3.4	49.1 ± 2.4	50.5 ± 4.0	57.1 ± 2.4
	20	45.8 ± 8.6	58.5 ± 3.7	63.6 ± 3.7	88.9 ± 4.0
**6**	5	25.0 ± 7.7	30.4 ± 8.5	26.6 ± 5.4	35.6 ± 1.1
	10	52.0 ± 7.8	37.0 ± 8.3	32.2 ± 3.1	30.7 ± 7.6
	20	74.8 ± 7.5	63.4 ± 7.1	78.7 ± 2.3	79.2 ± 3.7
**7**	5	25.7 ± 4.0	23.6 ± 0.8	19.6 ± 5.1	47.0 ± 8.0
	10	45.8 ± 5.9	34.4 ± 5.0	38.5 ± 5.7	89.0 ± 7.4
	20	60.2 ± 14.5	78.4 ± 2.6	62.2 ± 7.5	T.I.
**8**	5	11.4 ± 3.5	24.4 ± 1.5	18.3 ± 0.6	28.0 ± 8.2
	10	35.7 ± 7.1	28.4 ± 8.2	33.6 ± 9.1	45.7 ± 8.4
	20	51.6 ± 6.0	47.8 ± 5.1	45.5 ± 6.3	91.6 ± 6.9
**9**	5	26.4 ± 7.1	23.5 ± 4.8	32.0 ± 1.0	32.2 ± 9.8
	10	34.4 ± 3.0	36.9 ± 2.1	33.4 ± 10.7	70.6 ± 13.2
	20	55.8 ± 7.1	51.5 ± 7.5	56.3 ± 11.2	T.I.
**10**	5	14.0 ± 5.5	29.4 ± 6.0	47.0 ± 5.5	45.1 ± 10.0
	10	56.4 ± 8.6	41.4 ± 1.4	33.9 ± 11.6	93.6 ± 6.4
	20	70.2 ± 7.9	60.0 ± 5.9	84.7 ± 2.9	T.I.
**11**	5	29.2 ± 8.9	28.6 ± 5.0	18.4 ± 1.9	18.1 ± 2.2
	10	31.9 ± 8.9	31.5 ± 5.0	30.0 ± 2.2	40.5 ± 2.5
	20	51.5 ± 3.3	49.9 ± 2.1	60.4 ± 3.4	99.0 ± 1.6
**12**	5	35.8 ± 4.4	27.4 ± 6.0	15.4 ± 3.8	10.8 ± 2.0
	10	42.6 ± 8.3	39.2 ± 4.8	30.0 ± 2.7	47.4 ± 11.7
	20	85.8 ± 3.7	69.1 ± 7.9	80.9 ± 5.1	T.I.
**13**	5	46.8 ± 3.7	37.9 ± 12.2	22.1 ± 6.6	48.9 ± 9.2
	10	40.5 ± 13.5	58.9 ± 11.2	44.5 ± 2.5	77.7 ± 10.9
	20	68.0 ± 10.6	75.1 ± 6.2	73.1 ± 2.8	T.I.
**14**	5	29.7 ± 11.6	18.6 ± 2.2	16.1 ± 1.1	57.0 ± 11.1
	10	63.7 ± 8.6	43.4 ± 9.1	37.9 ± 11.8	97.0 ± 6.7
	20	74.5 ± 9.1	69.5 ± 5.3	57.4 ± 15.4	T.I.
**15**	5	33.3 ± 6.2	24.3 ± 6.4	17.2 ± 4.4	14.6 ± 6.5
	10	24.5 ± 11.2	44.0 ± 7.1	37.7 ± 2.4	13.0 ± 2.3
	20	33.6 ± 2.4	39.8 ± 10.1	37.0 ± 2.3	77.3 ± 6.3
**16**	5	37.3 ± 12.8	30.6 ± 4.4	40.8 ± 4.8	69.4 ± 13.7
	10	57.8 ± 8.9	52.4 ± 6.9	64.7 ± 1.8	81.6 ± 15.1
	20	78.3 ± 3.0	76.9 ± 4.6	87.6 ± 3.7	T.I.
**17**	5	19.5 ± 6.8	21.3 ± 6.7	19.6 ± 3.0	32.1 ± 10.2
	10	30.3 ± 5.2	29.3 ± 3.0	28.0 ± 1.4	44.2 ± 8.6
	20	47.0 ± 8.8	37.0 ± 3.9	38.9 ± 3.6	T.I.
**18**	5	9.8 ± 3.2	23.0 ± 9.3	28.7 ± 1.5	34.6 ± 11.3
	10	21.5 ± 14.8	33.0 ± 1.2	43.1 ± 2.9	84.1 ± 13.2
	20	26.8 ± 3.9	45.1 ± 15.3	59.9 ± 7.2	T.I.
Doxorubicin	5	82.8 ± 2.5	62.5 ± 1.8	73.2 ± 2.8	111.4 ± 2.8

Results are expressed as mean ± SEM (n ≥ 3). TI—total inhibition. Doxorubicin was used as a positive control.

**Table 2:** Effect of chalcone **16** on nitric oxide production by RAW 264.7, expressed as a percentage of inhibition of NO production.

Compound	Concentration (µM)	Time		
		0 h	6 h	14 h
Chalcone **16**	1.3	52.4 ± 10.1	19.0 ± 0.7 *	11.0 ± 1.1 *
	2.5	66.4 ± 3.6	24.8 ± 4.0 *	12.3 ± 2.0 *
Dexamethasone	5	58.1 ± 7.8	31.6 ± 1.8 *	5.9 ± 3.2 *

Results are expressed as mean ± SEM; n ≥ 3. Dexamethasone was used as a positive control. * *p* < 0.05.

## Data Availability

Not applicable.

## Sample Availability

Samples of the compounds are not available from authors.
